# Co-Utilization of Glucose and Xylose for Enhanced Lignocellulosic Ethanol Production with Reverse Membrane Bioreactors

**DOI:** 10.3390/membranes5040844

**Published:** 2015-12-03

**Authors:** Mofoluwake M. Ishola, Päivi Ylitervo, Mohammad J. Taherzadeh

**Affiliations:** 1 Swedish Centre for Resource Recovery, University of Borås, Allégatan 1, Borås 50190, Sweden; E-Mails: paivi.ylitervo@hb.se (P.Y.); mohammad.taherzadeh@hb.se (M.J.T.); 2 Department of Chemical and Polymer Engineering, Faculty of Engineering, Lagos State University, PMB 1012 Epe, Lagos, Nigeria

**Keywords:** detoxification, lignocellulosic ethanol, reverse membrane bioreactor, *Saccharomyces cerevisiae*, xylose fermentation

## Abstract

Integrated permeate channel (IPC) flat sheet membranes were examined for use as a reverse membrane bioreactor (rMBR) for lignocellulosic ethanol production. The fermenting organism, *Saccharomyces cerevisiae* (T0936), a genetically-modified strain with the ability to ferment xylose, was used inside the rMBR. The rMBR was evaluated for simultaneous glucose and xylose utilization as well as *in situ* detoxification of furfural and hydroxylmethyl furfural (HMF). The synthetic medium was investigated, after which the pretreated wheat straw was used as a xylose-rich lignocellulosic substrate. The IPC membrane panels were successfully used as the rMBR during the batch fermentations, which lasted for up to eight days without fouling. With the rMBR, complete glucose and xylose utilization, resulting in 86% of the theoretical ethanol yield, was observed with the synthetic medium. Its application with the pretreated wheat straw resulted in complete glucose consumption and 87% xylose utilization; a final ethanol concentration of 30.3 g/L was obtained, which corresponds to 83% of the theoretical yield. Moreover, complete *in situ* detoxification of furfural and HMF was obtained within 36 h and 60 h, respectively, with the rMBR. The use of the rMBR is a promising technology for large-scale lignocellulosic ethanol production, since it facilitates the co-utilization of glucose and xylose; moreover, the technology would also allow the reuse of the yeast for several batches.

## 1. Introduction

Ethanol production from lignocellulosic materials, such as woody biomass, forest, and agricultural residues, has the potential of reducing society’s dependence on fossil fuels [1,2,3]. Lignocellulosic materials consist of three main structural components: cellulose, hemicellulose, and lignin [4,5,6]. The hemicellulose fraction of hardwoods and agricultural residues, e.g., wheat straw, is dominated by xylose; hence, xylose utilization is essential for the successful fermentation of all the sugars into ethanol. However, the wild-type of yeast, *Saccharomyces cerevisiae*, which is commonly used for the fermentation of sugar to ethanol, cannot utilize xylose. Thus, the use of genetically-engineered *S. cerevisiae* for xylose uptake for the fermentation of xylose-rich biomass for ethanol production is one of the options that have been widely investigated [7,8,9]. On the other hand, the genetically-modified strain prefers glucose in a mixture of glucose and xylose, leading to the sequential utilization of sugars and, consequently, incomplete sugar utilization [10,11,12].

The cell retention strategy denoted as encapsulation has been reported to improve the xylose utilization and aids the *in situ* detoxification of the bioconvertible inhibitors [9,13,14]. Previous reports [9,13,15] show that the encapsulation of genetically-modified strains creates a sugar concentration gradient inside the tight agglomeration of cells. The glucose is consumed by the cells closer to the membrane of the capsules, thereby lowering the glucose concentration, which the inner cells closer to the core of the capsules acquire. Consequently, this improves the xylose uptake, thereby facilitating simultaneous sugar utilization. The encapsulated cells are in a microenvironment provided by the membrane layer of the capsules, a similar concept to the rMBR technology. However, encapsulating the cells is a laborious and time consuming task, since the process takes about 48 h to accomplish [16,17]. In addition, the capsules can easily disintegrate during the process with agitation. Moreover, complete xylose utilization was not achieved with the encapsulated cells [9].

Over the last decade, membrane bioreactors (MBRs) have had a conventional application in water and wastewater treatment [16,18,19]. In recent years, the MBRs have gained a wider application including its use in ethanol production. The technology has been used to e.g., enhance *in situ* detoxification of furfural [20] and to make it possible to perform continuous cultivations at high acetic acid concentrations [21]. MBRs have also been applied to allow optimum conditions in the hydrolysis and fermentation reactors in a recently developed simultaneous saccharification, filtration and fermentation (SSFF) process for lignocellulosic ethanol production [22]. The use of flat sheet membranes to contain and retain the cells can be advantageous over other cell retention methods such as encapsulation. The membrane modules are commercially available for use and could, thus, be a means of successfully creating several agglomerations of cells inside the panels, which will create the desired sugar concentration gradient in the agglomerates of the cells and eventually facilitate simultaneous utilization of both glucose and xylose. It will also facilitate *in situ* detoxification of the available lignocellulosic inhibitors in the medium to their less toxic derivatives and also create a possibility of instant cell reuse for several fermentation batches, even in substrates which contain particles. To our knowledge, the application of the flat sheet membranes in a reverse manner, wherein the yeast *S. cerevisiae* is inside the membrane panels for ethanol production, has not been reported in the literature.

This study investigated the use of integrated permeate channels (IPC) membranes in a reverse manner for lignocellulosic ethanol production, a technology referred to as rMBR. Simultaneous utilization of glucose and xylose was first investigated with the rMBR in a synthetic media. The rMBR was later evaluated in xylose-rich pretreated lignocellulosic material for sugar co-utilization and detoxification of the bioconvertible inhibitors.

## 2. Results and Discussion

The reverse membrane bioreactor (rMBR) provides a microenvironment for the yeast cells through the agglomeration of the yeast cells inside the membrane panels. The technology could facilitate a similar concentration gradient of sugars and inhibitors, as observed with the encapsulated cells [13] through the agglomerates of cells inside the inner matrix of the membrane panels. The agglomeration of cells will consequently facilitate the simultaneous utilization of glucose and xylose as well as *in situ* detoxification of the bioconvertible inhibitors. Moreover, the limitations of encapsulation such as long processing time, disintegration of capsules, and incomplete sugar utilization [9,16,17] necessitate the use of an improved technology, such as rMBR. The commercial availability of the membranes, as well as the ease of its application, makes the rMBR a preferred technology over encapsulation. The rMBR technology was investigated for simultaneous utilization of both glucose and xylose, as well as *in situ* detoxification of inhibitors in this study.

### 2.1. Performance of the Integrated Permeate Channel (IPC) Flat Sheet Membrane as rMBRs for Ethanol Production

**Figure 1:**
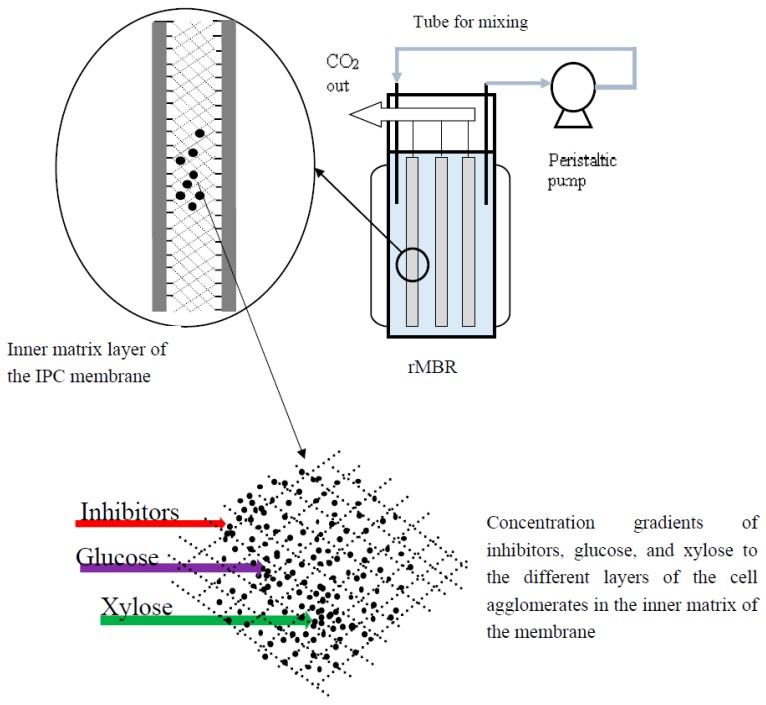
Schematic representation of the rMBR system configuration showing the IPC membrane panels’ inner matrix layers and the concentration gradient of glucose, xylose, and inhibitors into different layers of the yeast cell’s agglomerates inside the panels.

The IPC membranes were used in a novel way as rMBR with the genetically-modified yeast inside the membrane panels (Figure 1). The membranes performed well during their use, as the yeast inside the rMBR were able to ferment the available sugars to ethanol. Fouling is a major challenge in membrane applications [16,18]. However, the IPC membranes were successfully used for batch fermentations of the synthetic medium as well as the liquid fraction of the lignocellulosic hydrolyzate for up to eight days without fouling. The membranes were found to be reusable after the fermentation. The yeast cells inside the rMBR were also found to be metabolically active after the fermentation, as the cells were able to form colonies when plated. However, it was observed that after the panels had been used for two consecutive batch fermentations, there was a need for chemical cleaning and back-washing in order to get rid of all the cells entrapped inside the matrix of the membrane panels. This indicates that the rMBR can be used for a prolonged fermentation of lignocellulosic medium without any significant effect on the membranes or the cells.

### 2.2. Simultaneous Utilization of Glucose and Xylose in a Synthetic Medium with the rMBR

**Figure 2:**
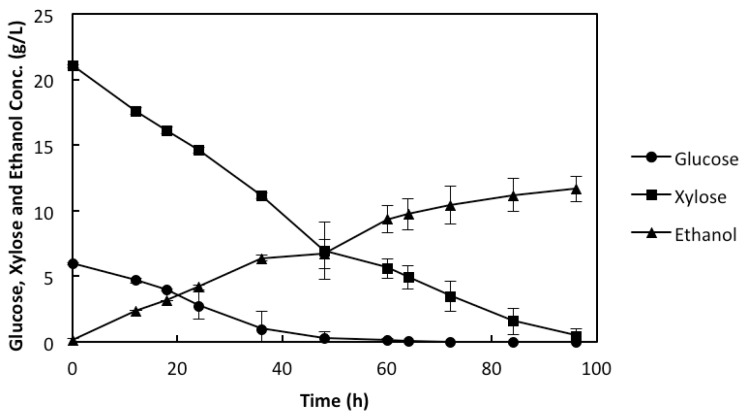
Concentration (g/L) profiles of glucose, xylose, and ethanol during the use of rMBR in the synthetic medium. The values presented are mean values of the two experiments, with error bars as the standard deviation between the two values.

In order to obtain a good ethanol yield and productivity during the fermentation of the xylose-rich lignocellulosic medium, it is important that the fermenting organism has an efficient uptake of xylose and conversion to ethanol. Xylose utilization by genetically-modified yeast strains has been previously reported to be dependent on glucose concentration, as low glucose concentration can facilitate xylose uptake [8]. Creating a sugar concentration gradient within the cell agglomeration has been reported to facilitate a controlled glucose supply to the different layers of the cell agglomerates which will, thus, facilitate the xylose uptake [13]. During the fermentation with the synthetic medium, it was observed that both the glucose and the xylose concentrations of 6 g/L and 21 g/L, respectively, decreased simultaneously (Figure 2), which indicates that there was a simultaneous uptake of both sugars during the fermentation. This shows that the inner fabric cross layer of the IPC membrane panels, which contained the genetically-modified yeast created the required agglomeration for the cells. Thus, there was a concentration gradient of sugars inside the cell agglomerates (Figure 1), which facilitated the xylose metabolism. Interestingly, after 96 h of fermentation, both the glucose and the xylose concentrations were completely consumed, and an ethanol concentration of 12 g/L was observed (Figure 2), which corresponds to 85.7% of the theoretical yield.

**Figure 3:**
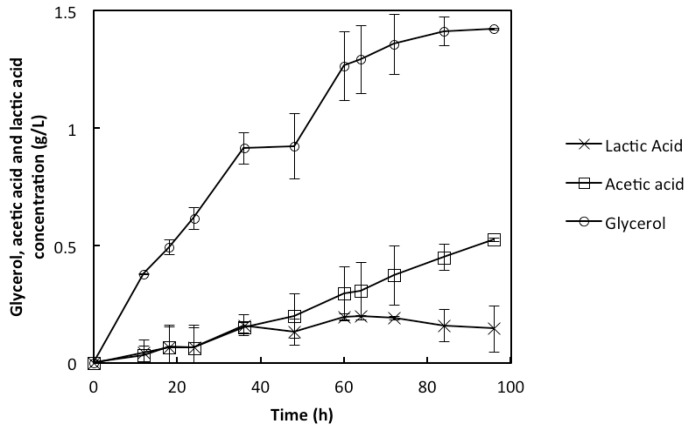
Concentration (g/L) profiles of glycerol, acetic acid, and lactic acid during the use of rMBR in the synthetic medium. Presented values are average values of two experiments, with error bars as the standard deviation between the two values.

The synthesis of glycerol during the yeast fermentations plays a significant role for its growth; glycerol maintains the intracellular redox balance as well as the osmoregulation with the cell’s external environment [23,24]. The glycerol concentration was observed to be very low during the fermentation, an indication that the cells inside the rMBR were able to maintain their metabolic activities throughout the fermentation, with very low glycerol formation and without having a significant effect on the ethanol yield. The highest glycerol concentration was 1.4 g/L during the fermentation, which lasted for 96 h (Figure 3); similar observation of low glycerol production with the specific strain has been reported [9]. Lactic acid concentration of approximately 0.2 g/L was measured at the end of the fermentation (Figure 3). This suggests that the chemical cleaning procedure used as a means of sterilizing and disinfecting the membrane panels in the reactor is effective in keeping contamination under control throughout the fermentation. It also indicates that the rMBR can successfully be used for prolonged fermentation without any contamination effect. Acetic acid is an inhibitor that is strongly pH dependent; it can be present in the medium as well as be a product of the fermentation [25]. Acetic acid concentration of only 0.5 g/L was produced by the end of the fermentation (Figure 3), which suggests that the acid was also produced during the fermentation with no direct effect on the fermentation process.

### 2.3. Co-Utilization of Sugars and *In Situ* Detoxification Using the Liquid Fraction of the Lignocellulosic Hydrolyzate with the rMBR

The performance of the rMBR was later investigated using the liquid fraction of the hydrolyzed wheat straw slurry. This was done in order to evaluate the performance of the rMBR in the real lignocellulosic medium. The supplied slurry of 14.9% SS was diluted to 10% SS with deionized water after which it was hydrolyzed. The 24 h enzymatic hydrolysis was performed at a temperature of 50 °C and a pH of 5.0. This increased the glucose concentration from 6.0 g/L up to 50.6 g/L, which indicates that the Cellic^®^ Ctec2 enzyme cocktail used was very effective for the cellulose hydrolysis. It was also observed that operating the enzymatic hydrolysis at optimum conditions is profitable for the lignocellulosic ethanol production, which is in agreement with a previous report on the benefits of performing the hydrolysis and fermentations at separate optimum conditions [22].

**Figure 4:**
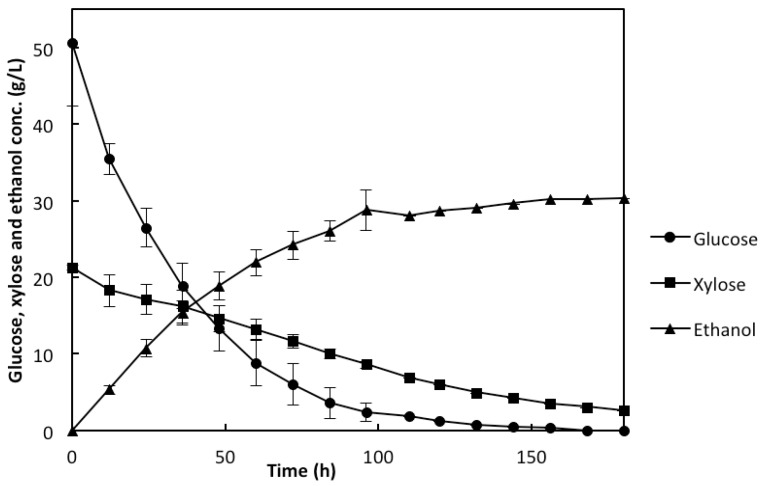
Concentration (g/L) profiles of glucose, xylose, and ethanol during the use of rMBR in the liquid fraction of the prehydrolyzed pretreated wheat straw used as xylose-rich lignocellulosic material. The values presented are mean values of two experiments, with error bars as the standard deviation between the two values.

During the fermentation with the rMBR, despite the presence of the inhibitors; e.g., acetic acid, HMF, and furfural in the medium, the co-utilization of glucose and xylose was observed (Figure 4). The glucose was consumed faster than the xylose, as the 50.1 g/L glucose was completely depleted within 132 h while 2.6 g/L of the xylose remained, out of the initial concentration of 21.2 g/L after the fermentation that lasted for 180 h (Figure 4). This corresponds to 87.7% xylose utilization. It was observed that the xylose uptake by the genetically-modified yeast encased in the membrane panels was not affected by the high initial glucose concentration of 50.1 g/L. This suggests that the rMBR resulted in a higher xylose uptake compared to the study performed by the encapsulated yeast used in a SSFF process, where a 80% xylose uptake was reported at an initial glucose concentration of 36 g/L, while only 36% xylose uptake was reported at an initial glucose concentration of 53.5 g/L due to catabolite repression [9].

The observations from this study indicate that a high initial glucose concentration in the medium does not really affect the xylose uptake by the genetically-modified strain as much as the fermentation process used does. Creating a microenvironment for the cells by having the cells inside the IPC membranes facilitated the co-utilization and higher xylose uptake despite the initial glucose concentration. The possibility of achieving the desired concentration gradient due to the agglomeration of cells in the panels facilitated the improved xylose uptake.

**Figure 5:**
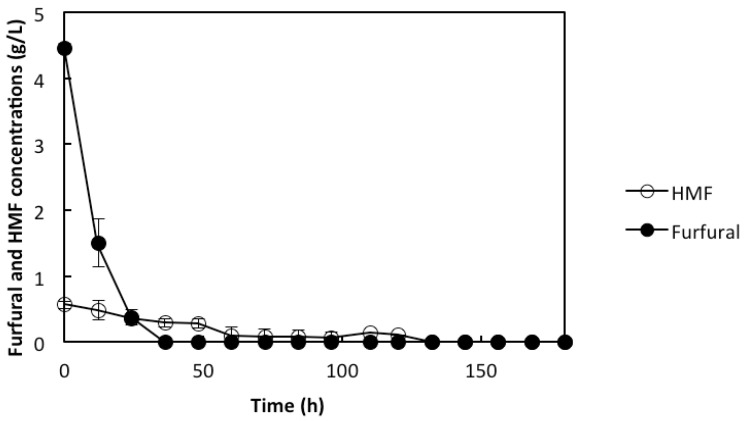
Concentration (g/L) profiles of furfural and HMF during the use of rMBR in the liquid fraction of prehydrolyzed pretreated wheat straw used as xylose-rich lignocellulosic material. Presented values are average values of two experiments, with error bars as the standard deviation between the two values.

*In situ* detoxification of furfural and HMF was also observed. Within 36 h of fermentation, the initial furfural concentration of 4.5 g/L was completely converted (Figure 5), while the initial HMF concentration of 0.6 g/L was completely converted within 60 h (Figure 5). Although HMF has a lesser inhibitory effect compared to furfural, its rate of conversion has been reported to be slower than that of furfural [26,27] as observed in this study. This observation suggests that the agglomeration of cells in the panels, which created the concentration gradient of the streams reaching the cells, does not only facilitate the co-utilization of the glucose and xylose but also helps with the *in situ* detoxification. The cells close to the outer layer of the agglomerates detoxified the inhibitors, hence, improving the rate of their conversion (Figure 1). This observation is in agreement with a previous report on *in situ* detoxification of the bioconvertible inhibitors [14]. The rate of the *in situ* detoxification with the rMBR was much faster than an earlier reported rate with the particular strain when encapsulation was used with the SSFF process. It was reported that it took up to 72 h for both HMF and furfural to be completely converted even though a cell concentration of 6 g/L was used [9]. With the use of the rMBR in this study, the ethanol concentration of 30.3 g/L was observed at the end of the fermentation, which lasted for 180 h (Figure 4). This corresponds to 83% of the theoretical ethanol yield. It can, thus, be stated that the rMBR with the IPC membrane panels is beneficial for the co-utilization of glucose and xylose utilization as well as for the *in situ* detoxification of the bioconvertible inhibitors, which are important factors for an efficient and successful lignocellulosic ethanol production.

## 3. Experimental Section

### 3.1. Lignocellulosic Material

Wheat straw, an agricultural residue from a Swedish farm was used in the experiments. It is a xylose-rich lignocellulosic biomass. The biomass was chemically pretreated with dilute H_2_SO_4_ (0.3%–0.5%) at 185 °C for 8 min at SEKAB E-Technology (Örnsköldsvik, Sweden). It was supplied as a pretreated slurry with pH of 1.9, 14.9% suspended solids (SS), and 22.2% total solids. The slurry was stored in a cold room at 5 °C until use. The composition of the liquid fraction of the slurry was analyzed by the high-performance liquid chromatography (HPLC) and presented in Table 1. The solid fraction of the slurry was characterized according to the NREL protocols [28]; it contained 43.6% ± 0.5% cellulose, 34.8% ± 0.1% acid insoluble lignin (AIL), 5.1% ± 0.1% acid soluble lignin (ASL), and 39.9% ± 0.2% total lignin. The hemicellulose fraction was completely fractionated into monomeric sugars during the pretreatment. The theoretical ethanol yield was calculated based on 0.51 g/g of the glucose and xylose available in the liquid fraction of the enzymatically-hydrolyzed slurry.

**Table 1:** Composition of the liquid fraction of the supplied pretreated wheat straw (slurry), used as xylose-rich lignocellulosic biomass with 14.9% suspended solids.

Component		Concentration (g/L)
Monomeric sugars	Xylose	33.4
	Glucose	8.5
	Mannose	1.5
	Arabinose	4.9
	Galactose	3.1
Inhibitors	Acetic acid	8.9
	HMF	1.1
	Furfural	9.2

### 3.2. Enzymes and Yeast Strain

The Cellulase Cellic^®^ Ctec2 enzyme (Novozymes, Bagsvaerd, Denmark) was used for the enzymatic hydrolysis. The enzymatic activity was measured as 185 FPU/mL activity and was determined according to the NREL method [29]. A genetically-engineered strain of *Saccharomyces cerevisiae* (T0936) was used in all the experiments. The yeast was maintained at 4 °C on yeast extract, peptone, and dextrose (YPD) agar plates, containing 20 g/L agar, 10 g/L d-glucose, 10 g/L d-xylose, 10 g/L yeast extract, and 20 g/L peptone. Prior to the fermentations, 200 mL yeast pre-culture in the YPD growth medium, containing 25 g/L d-glucose, 25 g/L xylose, 20 g/L peptone, and 10 g/L yeast extract was prepared by cultivations performed in two 250 mL Erlenmeyer flasks with 100 ml medium in each. The flasks were incubated in a shake-bath (Grant OLS 200, Grant Instrument Ltd., Royston, UK) at 121 rpm and 30 °C for 48 h.

### 3.3. Cell Cultivation for the rMBR

In order to achieve a high cell concentration inside the membrane panels, the 200 mL pre-culture was inoculated into a 2.5 L bioreactor (Infors AG107504, Minifors, Bottmingen, Switzerland) at 30 °C and pH 5.0, using a YPD growth medium consisting of 17 g/L d-glucose, 33 g/L d-xylose, 20 g/L peptone, and 10 g/L yeast extract. Silicone antifoam (1 g/L) and 3.5 g/L KH_2_PO_4_ were added. The cultivation was aerated at 4.0 vvm for 42 h to produce a cell concentration of 5 g/L per intended fermentation volume of 3 L. After the cell cultivation, the cells were harvested by centrifugation at 4500 rpm for 5 min and concentrated into 150 mL, thus, making a cell concentration of 100 g/L per membrane volume of 150 mL

### 3.4. Flat Sheet Integrated Permeate Channels (IPC) as the rMBR

The IPC membrane panels were developed and produced by the Flemish Institute for Technological Research (Vito NV, Boeretang, Mol, Belgium). The polymeric membrane panels consist of double membrane layers that consist of an integrated permeate channels, interposed to the two membrane layers. The IPC membrane was manufactured of PES/PVP with a pore size of 0.3 μm. The IPC membranes are unique for their sturdiness and ability to withstand high pressure differences during the filtration and backwashing [30]; this property qualifies the membrane for application as rMBR. Each membrane panel had a total membrane area of 0.0252 m^2^, which was available for the filtration and can take a volume of about 50 mL Three membrane panels were placed in parallel inside a 4.0 L bioreactor (Webant BE0076, Belach Bioteknik AB, Skogås, Sweden).

The membrane panels cannot be sterilized by autoclavation; hence, they were chemically cleaned and disinfected according to the following procedures before each fermentation run. First, the membrane panels were immersed in a two percent NaOH solution at 50–80 °C for 30 min, after which they were rinsed with deionized water. Then, 1% phosphoric acid solution was applied to the panels for 30 min and then rinsed with deionized water. Thereafter, the panels were disinfected with 200 ppm NaOCl solution and rinsed with sterile deionized water before the fermentation run. Finally, the concentrated cultivated cells described earlier were aseptically transferred into the membrane panels, resulting in 5 g/L dry weight of the yeast cells that were added to the rMBR.

### 3.5. Configuration of the Reverse Membrane Bioreactor (rMBR)

The schematic configuration of the rMBR is shown in Figure 1. The genetically-modified yeasts cells were added inside the membrane panels, which were submerged in the medium inside the reactor (Figure 1). The inner matrix layer of the membrane panels created a microenvironment for the yeast cells and resulted in several agglomerations of the cells. The yeast cells inside the panels acquired substrate and nutrients when the medium diffuses through the membrane panel. The metabolites also diffuse through the membrane panels into the medium.

### 3.6. Synthetic Medium Fermentation with the rMBR

The performance of the genetically-modified yeast cell inside the rMBR was first investigated in 3 L synthetic medium containing glucose and xylose, equivalent to that present in the 10% suspended solids (SS), corresponding to 6 g/L glucose and 21 g/L xylose. The medium was supplemented with 2 g/L (NH_4_)_2_SO_4_, 0.35 g/L KH_2_PO_4,_ and 1 g/L yeast extract; 0.6 g/L silicone antifoam was also added. The three membrane panels containing the yeast cells were submerged in the fermentation medium inside the bioreactor. The fermentation was performed anaerobically at a temperature of 30 °C and a pH of 5.0. A marprene process tube (internal diameter 8.0 mm, thickness 2.4 mm and outer diameter 12.6 mm, 902.0080.24, Watson Marlow, Falmouth, England) was used for circulating the liquid inside the bioreactor with a peristaltic pump (323DU/D Watson Marlow, Falmouth, England) at a flow rate of 0.6 L/min and thereby mixing the medium. Fermentation was carried out for 96 h, and samples were taken every 12 h from the medium to monitor the sugar consumption and the metabolite production.

### 3.7. Fermentation of the Liquid Fraction of the Hydrolyzed Pretreated Wheat Straw with the rMBR

Enzymatic hydrolysis of the pretreated wheat straw was carried out at a temperature of 50 °C, pH of 5.0, and agitation of 700 rpm for 24 h. Substrate and enzyme loading of 10% SS and 12 FPU/g SS, respectively, was used. Thereafter, the hydrolyzate was centrifuged aseptically (5000 rpm, 5 min) in order to separate the liquid fraction from the solid residue. The liquid fraction containing the sugars and the inhibitors was later used for the fermentation, in a similar way as the synthetic medium described above, with freshly cultivated genetically-modified yeast inside the membrane panels. All of the experiments were performed in duplicates, with error bars showing the standard deviation.

### 3.8. Analytical Methods

Cellulose, hemicellulose and lignin contents of the solid fraction of the slurry was determined according to the NREL protocols [28]. The solid and liquid fractions of the slurry were separated with a centrifuge at 4000 × *g* for 5 min. The solid fraction was washed with 40 mL deionized water to a neutral pH and then freeze-dried (Labconco, Kansas City, MO, USA) at −52 °C until its moisture content was less than 10%. Two steps hydrolysis was later performed on the dried solid fraction: first with 72% H_2_SO_4_ in a shaker water bath at 30 °C for 60 min, and later with 4% H_2_SO_4_ in an autoclave at 121 °C for 60 min. The ASL was determined on the liquid portion of the hydrolyzate with a UV spectrophotometer (Libra S60, Biochrom, Cambridge, UK) at 283 nm. The AIL was gravimetrically determined as the residual solid after the hydrolysis, corrected with the ash content. The ash content was determined in the muffle furnace at 575 °C overnight. The hydrolysis liquid was analyzed by HPLC for the monomeric sugars.

The sugars and the metabolic products were analyzed using a HPLC (Walters 2695, Walters Corporation, Milford, CT, USA). A hydrogen-based column (Aminex HPX-87H, Bio-Rad, Hercules, CA, USA) at 60 °C and 0.6 mL/min 5 mM H_2_SO_4_ as eluent was used for the glucose, furans, carboxylic acids, ethanol, lactic acid, and glycerol. Mannose, glucose, galactose, xylose, and arabinose were analyzed using the Aminex HPX-87P column (Bio-Rad) at 85 °C and 0.6 mL/min ultrapure water as an eluent. A UV absorbance detector (Walters 2487), operating at 210 nm wavelength, was used in a series with a refractive index (RI) detector (Walters 2414).

## 4. Conclusions

Integrated permeate channel (IPC) flat sheet membranes were examined as a reverse membrane reactor (rMBR) for lignocellulosic ethanol production. The rMBR was investigated for co-utilization of glucose and xylose utilization as well as for *in situ* detoxification of inhibitors. The synthetic medium was initially investigated, followed by using the liquid fraction of the enzymatically hydrolyzed pretreated wheat straw as a xylose-rich media. The IPC membrane panels containing the yeast cells were successfully used for the batch fermentation lasting for up to eight days without fouling. With the rMBR, complete xylose utilization, together with 86% of the theoretical ethanol yield, was observed when the synthetic medium was used. Its usage with the pretreated wheat straw resulted in 87% xylose utilization and complete *in situ* detoxification of furfural and HMF within 36 h and 60 h, respectively; a final ethanol concentration of 30.3 g/L equivalent to an ethanol yield of 83% of the theoretical value was obtained. The use of the rMBR with the yeast cells inside the membranes could be a promising technology in the future for large-scale lignocellulosic ethanol production.
